# Observation of juvenile Eastern Blue Groper (*Achoerodus viridis*) on remnant oyster reefs in New South Wales, Australia

**DOI:** 10.1002/ecy.3824

**Published:** 2022-09-22

**Authors:** Christopher Pine, Katherine Erickson, Paul E. Gribben, Will F. Figueira

**Affiliations:** ^1^ School of Life and Environmental Sciences University of Sydney Syndey New South Wales Australia; ^2^ Centre for Marine Science and Innovation, School of Biological, Earth and Environmental Sciences University of New South Wales Sydney New South Wales Australia; ^3^ Sydney Institute of Marine Science Mosman New South Wales Australia

**Keywords:** charismatic megafauna, conservation, demography, estuarine ecosystem, intertidal reef, life history, recruitment, temperate reef, threatened species

Understanding the life cycle and the demography of individual species is critical for the management of threatened populations and integral to understanding the ecology of various ecosystems and habitats (Kimirei et al., 2015; Perez & Munch, 2010). This knowledge is particularly critical for fish species, as the maintenance of populations and biodiverse assemblages is heavily influenced by larval supply, post‐settlement processes, and demographic processes such as recruitment (Caley et al., 1996). The successful recruitment of a juvenile fish into the adult population is strongly determined by individual size, particularly in marine environments, where selection pressures favoring larger individuals are approximately five times the strength of the same pressure on terrestrial taxa (Perez & Munch, 2010). As such, habitats that provide the necessary resources to support high rates of juvenile growth are important for the survival of juveniles and their recruitment into the wider population (Lefcheck et al., 2019; Perez & Munch, 2010). Marine habitats that are able to support this can have a greater density and abundance of juvenile species (e.g., seagrass meadows, kelp forests, mangroves) and are termed “nursery habitats” (Lefcheck et al., 2019).

*Achoerodus viridis* (Eastern Blue Groper) is an iconic fish species in eastern Australia, and particularly in the state of New South Wales (NSW) where it is the state marine emblem due to its popularity and social importance. This species is listed as “Near Threatened” by the International Union for Conservation of Nature (IUCN) due to the loss of key habitat and historical overfishing, and is a protected species in the states of NSW and Victoria (Choat & Pollard, 2010). They belong to the Labridae family of fish and are a long‐lived hermaphroditic species, which start out as female juveniles with an initial‐phase color of pinky brown (Figure 1a), growing to sexually mature females (Figure 1b) before transitioning to an adult male form in which they take on their iconic blue color (Figure 1c; Gillanders, 1995). *A. viridis* have a biphasic life cycle, starting out in a presettlement planktonic larval phase before transitioning to a post‐settlement juvenile/adult phase. *A. viridis* larvae often settle onto seagrass meadows, *Ecklonia* (kelp) forests, or in‐shore rocky reef habitats and occupy these habitats throughout their juvenile phase (Gillanders, 1997b; Gillanders & Kingsford, 1996). Mature *A. viridis* often emigrate from these juvenile habitats and recruit into adult populations on exposed costal reefs or habitats near the entrance of estuaries (Gillanders, 1997b; Gillanders & Kingsford, 1996). This movement is most likely to be a response to post‐settlement selection pressures, as the resources in the juvenile habitat become inadequate to support the individual, or other factors such as predation pressures become more prominent (Gillanders, 1997a, 1997b).

**FIGURE 1:**
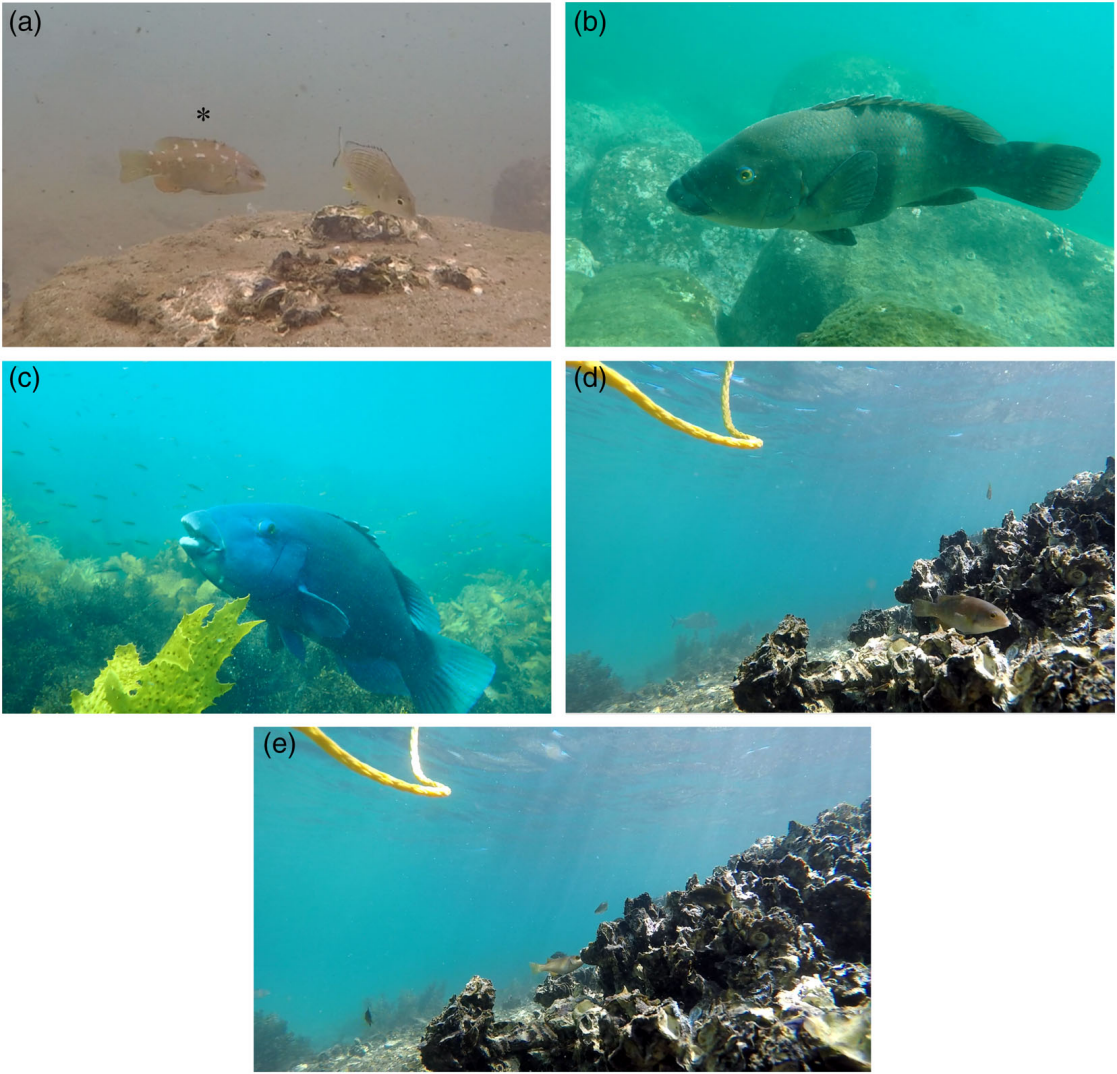
*Achoerodus viridis* at various life stages (a–c) and *A. viridis* interacting with *S. glomerata* oyster reefs (d, e). (a) Juvenile on an oyster reef (indicated by *). (b) Adult female on a rocky reef. (c) Adult male in a kelp (*Ecklonia radiata*) forest. (d) Juvenile resting on the oyster matrix (Bermagui). (e) Juvenile feeding on the oyster reef (Bermagui). Images (b) and (c) courtesy of Dione Deaker.

These in‐shore estuarine habitats are particularly vulnerable to the increasing pressures of climate change and other anthropogenic impacts such as eutrophication and decreasing water quality; leading to the degradation or loss of these key habitats (Lotze et al., 2006). This poses a threat to the long‐term stability of many *A. viridis* metapopulations, as the loss of key recruitment habitats may decrease the ability of estuarine and coastal systems to support an abundant population of *A. viridis*. As such, furthering our understanding of the life history of *A. viridis* by identifying critical recruitment and juvenile habitats will ensure the long‐term stability of the *A. viridis* population. Here we report on the novel use of oyster reefs on the southeast coast of Australia by juvenile *A. viridis* (Eastern Blue Groper; hereafter referred to as blue groper).

Juvenile blue gropers were observed as part of a broader study that conducted seasonal (summer and winter) remote underwater video surveys of fish assemblages on four remnant oyster reefs in NSW (Bermagui, Crookhaven, Towra Point, Port Hacking; Pine et al. unpublished manuscript). For each video, the MaxN (the maximum number of individuals of a given species observed in a single frame throughout a video) of each species was recorded to obtain a measure of relative abundance (Schobernd et al., 2013). At the sites where *A. viridis* were recorded (Bermagui, Crookhaven, Towra Point), observations of juvenile blue groper were common, occurring in 29.8% of videos taken during summer (37 out of 124), although the frequency varied by location with observations in 56%, 65%, and 3% of all videos at Crookhaven, Bermagui, and Towra Point, respectively. Based on ranked MaxN values (with hyperabundant shoaling species *Hyperlophus vittatus*, *Ambassis jacksoniensis*, and *Atherinomorus vaigiensis* removed) juvenile *A. viridis* were moderately common at Bermagui and Crookhaven, ranking 7th out of 17 total ranks and 8th out of 23 respectively (Figure 2a,b). They were much less common at Towra Point, ranking 16th out of 17 ranks (Figure 2c).

**FIGURE 2:**
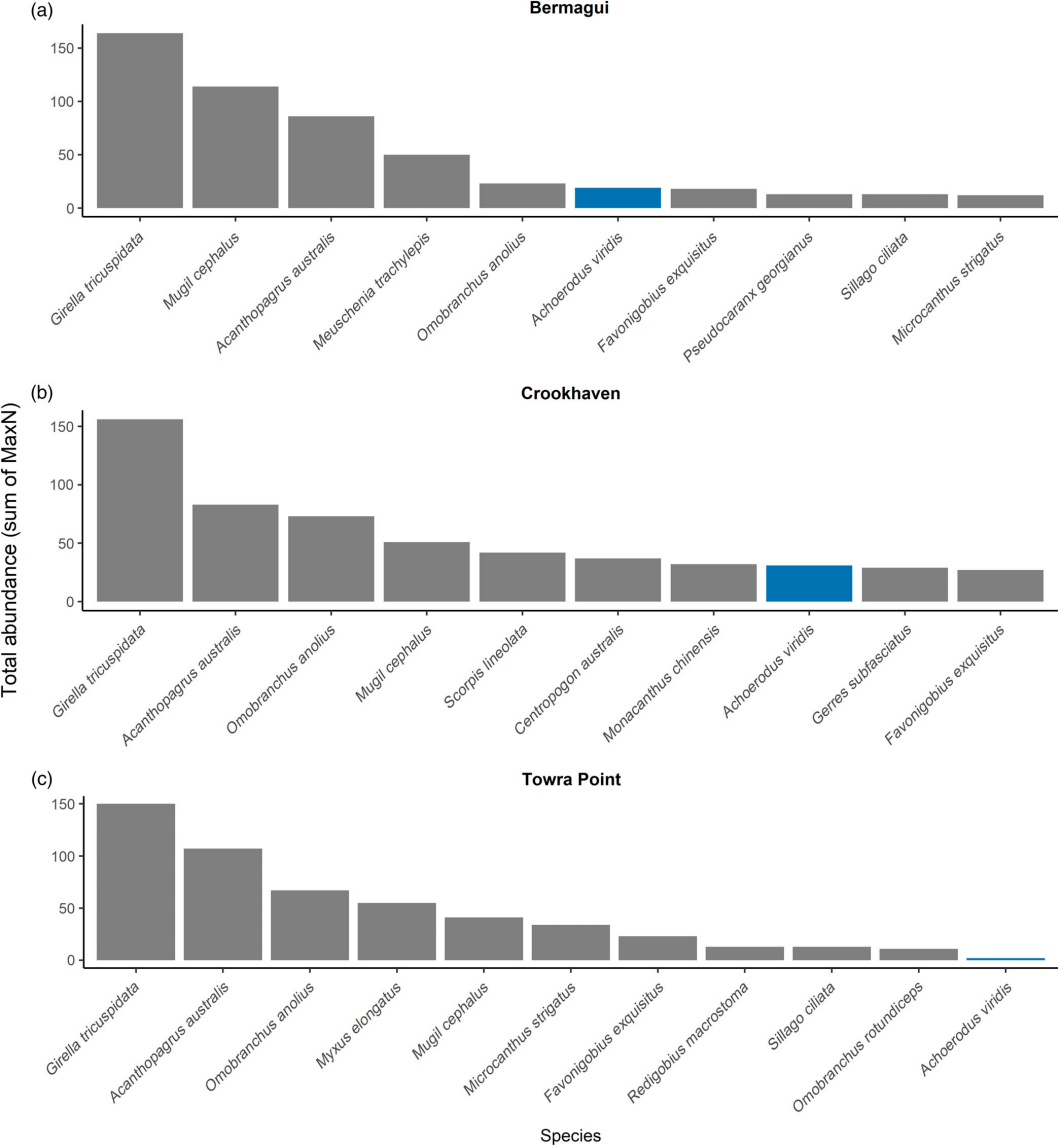
Total abundance (sum of MaxN) of the top 10 most abundant species (and *Achoerodus viridis*, which was not in the top 10) at each of the sites where in this study *A. viridis* was observed.

The frequency of their occurrence at Bermagui and Crookhaven gives an indicator of their abundance on threatened oyster reefs and suggests that this is not just a one‐off vagrancy event from traditional habitats (e.g., kelp forests, seagrass meadows, estuarine rocky reefs), but that oyster reefs may be playing a critical, yet to date underappreciated, role in the ontogenetic movement of this species in these regions. The lack of any individuals at Port Hacking and low numbers of individuals at Towra Point is interesting, given the fact that these two estuaries are more urbanized (located within Sydney) and more degraded than the other two systems (Reid, 2020). Urbanized systems are more likely to have limited recruitment due to increased levels of habitat fragmentation/loss and decreased water quality, in addition to lower levels of fish stock due to increased fishing pressures (Taylor & Suthers, 2021). This could potentially be why we did not observe the same level of abundance of *A. viridis* in these estuaries compared with Bermagui and Crookhaven.

In the majority of observations, individuals were directly interfacing with the oyster reef matrix, either using the reef for protection, or feeding on small invertebrates and epifauna found on the oyster shells (Figure 1d,e). The observation of individuals using the matrix for protection is particularly important as the three‐dimensional complexity of oyster reefs and other hard‐structured habitats can reduce predation pressures and the impacts of the hydrodynamic environment on individuals, both of which have deleterious impacts on the mortality rate and demography of juveniles (Forrester, 1990; Johnson, 2006). Furthermore, the observation of this frequent foraging behavior suggests that juvenile *A. viridis* are utilizing these threatened oyster reefs as a source of food, potentially enhancing their growth rate and recruitment success.

Notably, no adult blue gropers were observed during the study. The lack of adults present on these reefs is not surprising, as juvenile *A. viridis* are known to emigrate to more exposed coastal habitats as they mature due to the greater availability of resources and favorable conditions (Gillanders, 1997b). However, this highlights the key role that oyster reefs may play within the ontogenetic movements of *A. viridis*.

This observation of an iconic and threatened species using threatened oyster reefs as a juvenile habitat is significant, not only because it enhances our understanding of *A. viridis* and their ontogenetic movement, but because of the opportunity that this presents for the engagement and funding of ongoing and future oyster reef restoration projects. Over the past century, nearly 85% of all oyster reefs have been lost worldwide due to various anthropogenic impacts (Beck et al., 2011). In Australia, reefs consisting of the two endemic oyster species, *Saccostrea glomerata* (Sydney Rock Oyster) and *Ostrea angasi* (Southern Mud Oyster) are considered functionally extinct, and while there is significant momentum for restoration efforts within Australia, such work is costly and lengthy, and maintaining interest is likely to be difficult (Beck et al., 2011; Schrobback et al., 2014). The identification of threatened oyster reefs as a key habitat for threatened juvenile blue gropers is likely to buoy these efforts and increase public support, a phenomenon often observed when iconic species are used in support of conservation efforts (Lee et al., 2015).

The observations we report here introduce the possibility that the decline in a potential key habitat (oyster reefs) may have been an unforeseen barrier to efforts to stabilize blue groper populations in the past. If this is the case, restoration and conservation of these threatened oyster reefs may enhance ontogenetic movement opportunities for the species by providing key juvenile habitats, which could further aid in the long‐term stability and protection of blue groper populations. Although beyond the scope of this paper, further studies into the recruitment and demography of juvenile *A. viridis* on threatened oyster reefs is needed to enhance our understanding of its life history and to better predict the impact that oyster reef restoration may have on the population status of this iconic species.

## CONFLICT OF INTEREST

All authors declare no conflicts of interest.

## Data Availability

Fish abundance data, images, and code used to analyze the data and create the figures are freely available via two open access repositories: CPineMarineEcology (2022) on Zenodo at https://doi.org/10.5281/zenodo.6687723, and Pine et al. (2022) on the Sydney eScholarship Repository at https://hdl.handle.net/2123/28891.
